# Short-term oral pre-exposure prophylaxis against HIV-1 modulates the transcriptome of foreskin tissue in young men in Africa

**DOI:** 10.3389/fimmu.2022.1009978

**Published:** 2022-11-18

**Authors:** Stefan Petkov, Carolina Herrera, Laura Else, Limakatso Lebina, Daniel Opoka, Thabiso B. Seiphetlo, Azure-Dee AP. Pillay, Susan Mugaba, Patricia Namubiru, Geoffrey Odoch, Andrew S. Ssemata, Jennifer Serwanga, Pontiano Kaleebu, Emily L. Webb, Saye Khoo, Neil Martinson, Clive M. Gray, Julie Fox, Francesca Chiodi

**Affiliations:** ^1^ Department of Microbiology, Tumor and Cell Biology, Karolinska Institutet, Stockholm, Sweden; ^2^ Department of Infectious Disease, Imperial College London, London, United Kingdom; ^3^ Department of Molecular and Clinical Pharmacology, University of Liverpool, Liverpool, United Kingdom; ^4^ Perinatal HIV Research Unit, University of the Witwatersrand, Johannesburg, South Africa; ^5^ Medical Research Council/Uganda Virus Research Institute and London School of Hygiene & Tropical Medicine Uganda Research Unit, Entebbe, Uganda; ^6^ London School of Hygiene & Tropical Medicine, London, United Kingdom; ^7^ Division of Molecular Biology and Human Genetics, Biomedical Research Institute, Stellenbosch University, Cape Town, South Africa; ^8^ Life Sciences & Medicine, King’s College London, London, United Kingdom

**Keywords:** emtricitabine tenofovir, pre-exposure prophylaxis PrEP, transcriptomes, inflammation, foreskin, mitochondrial function

## Abstract

Whilst short-term oral pre-exposure prophylaxis (PrEP) with antiretroviral drugs in men who have sex with men has shown protection against HIV-1 infection, the impact of this regimen on the *in vivo* foreskin transcriptome is unknown. We collected foreskin tissue after voluntary medical male circumcision from 144 young men (72 from Uganda and 72 from South Africa) randomized to one to two doses of either oral tenofovir (TFV) disoproxil fumarate (FTC-TDF) or tenofovir alafenamide (FTC-TAF) or no drug (untreated controls). This novel approach allowed us to examine the impact of short-term oral PrEP on transcriptome of the male genital tract. A single dose of FTC-TDF did not affect the foreskin transcriptome in relation to control arm, however one dose of FTC-TAF induced upregulation of four genes *AKAP8*, *KIAA0141*, *HSCB* and *METTL17*. Following two doses of either FTC-TDF or FTC-TAF, there was an increase in 34 differentially expressed genes for FTC-TDF and 15 for FTC-TAF, with nine DEGs in common: *KIAA0141*, *SAFB2*, *CACTIN*, *FXR2*, *AKAP8*, *HSCB*, *CLNS1A*, *DDX27* and *DCAF15*. Functional analysis of differentially expressed genes revealed modulation of biological processes related to mitochondrial stress *(KIAA0141, HSCB and METTL17)*, anti-viral and anti-inflammatory pathways (*CACTIN* and *AKAP8)*. Our results show that short-course on-demand oral PrEP in men modulates genes in foreskin tissue which are likely unfavorable to HIV acquisition and replication. We also describe an upregulated expression of genes involved in diverse mitochondria biology which may potentially result in worsened mitochondria-related. These results warrant further studies to assess the role of short-course and prolonged oral PrEP on biological processes of the foreskin mucosa.

## Introduction

Prevention of infection by human immunodeficiency virus (HIV) has become an achievable goal with the implementation of pre-exposure prophylaxis (PrEP) by administration of emtricitabine (FTC) with tenofovir (TFV) disoproxil fumarate (FTC-TDF) or tenofovir alafenamide (FTC-TAF). The effectiveness of TDF as daily and on-demand PrEP has been demonstrated in men who have sex with men (MSM) (1). The newer drug combination FTC-TAF, has not been evaluated for on-demand use but has shown high efficiency as a daily regimen (2). To date, a dosing regimen for insertive sex had not been identified. The combined HIV Adolescent PrEP and Prevention Study (CHAPS, NCT03986970) sought to identify a dosing regimen for insertive sex and to compare the efficacy of TDF and TAF (3). Although highly effective at inhibiting HIV replication, FTC-TDF administration results in high levels of TFV in plasma that have been associated with adverse events such as renal tubulopathy (1, 4, 5). The prodrug TAF presents a safer alternative (6) as it only accumulates intracellularly thereby reducing the exposure to high levels of TFV, which have been linked to nephrotoxicity (7). The higher level of TAF accumulation in PBMCs has also led to the suggestion that HIV protection may be higher with TAF.

Despite being systemically safe and well-tolerated, the impact of oral FTC-TAF/TDF on local tissues harboring HIV target cells, and particularly the genital tract, remains largely unexplored. Hladik et al. recently conducted a systematic evaluation of topical application of 1% TFV gel on the rectal mucosa (8); in this study, the RNA transcriptome was analyzed in biopsies taken proximal to the anal margin after seven consecutive daily applications of the gel after which transcript levels were compared to the untreated tissues biopsied at enrollment. The results revealed several modulated genes after a single application and at day seven 505 genes were suppressed and 137 induced. The authors concluded that due to the wide range of modulated genes, treatment can potentially affect mucosal immune homeostasis, mitochondrial function, and regulation of epithelial cell differentiation and survival. Another study investigating the effect of eight daily rectal applications of 1% TFV gel in MSM using a proteomic approach identified more than 250 modulated proteins with 40 unique functions in rectal sponge eluates (9). The protein signature associated with TFV use was related to the structural integrity of the epidermal barrier; these results suggested that exposure to TFV-based microbicides may negatively affect epidermal function.

Unlike topical application, oral administration results in up to 100-fold lower levels of active drug in mucosal tissue (10, 11). In a recent study Hughes et al. examined the effect of oral FTC-TDF administration on the transcriptome of a wide array of samples from different tissues including gut, female genital tract and PBMCs (12). Interestingly, oral administration of a daily FTC-TDF dose in women for up to 36 months did not affect gene expression in the vagina or PBMCs (13). After treatment for two months, a similar lack of transcriptome changes was observed in PBMCs; differentially expressed genes (DEGs) were however observed in rectal (13 DEGs) and duodenal (251 DEGs) tissues (12). The modulated genes were mainly associated with type I/III IFN pathways, which could be interpreted as increased innate immune readiness, enhancing the antiviral preventive efficacy of treatment, but also as a source of chronic inflammation upon prolonged administration.

The effect of short-term on-demand PrEP using FTC-TAF/TDF remains to be determined. As sexual transmission of HIV-1 occurs at the mucosal surface it is of utmost importance to understand how different PrEP regimens affect the genital mucosa. To fill this knowledge gap, in this study we used foreskin samples obtained after medical male circumcision (MMC) in the CHAPS trial to study the changes in gene expression after one to two days of FTC-TAF and FTC-TDF, given as short-dose PrEP.

## Materials and methods

### Specimen collection

**Figure 1:**
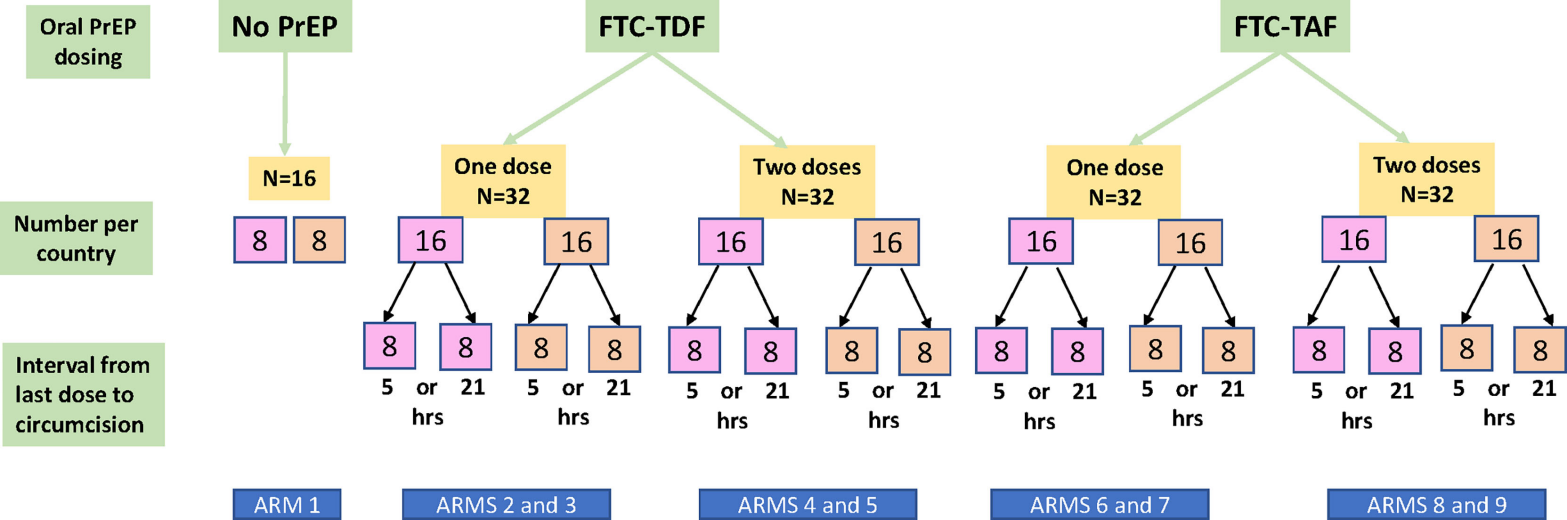
Flow diagram of participants in the CHAPS trial. The participants are divided according to PrEP drug (FTC-TDF or FTC-TAF), number of PrEP doses (one or two) and interval between last dose and circumcision (5 or 21 hours). The number of included participants from South Africa is shown in the pink boxes and for Uganda in the orange boxes. Arm 1 consists of control individuals who did not receive PrEP. Each arm includes an equal number of participants from South Africa and from Uganda.

The study design, methodology and recruitment pathways of the CHAPS trial have been previously reported (3). Foreskin tissue was collected at voluntary MMC clinic at Chris Hani Baragwanath Academic Hospital in Soweto (South Africa) and Entebbe Regional Referral Hospital and the Uganda Virus Research Institute (UVRI) clinic (Uganda) after PrEP was administered. The median age of study participants in Uganda was 19 years and in South Africa 19.5 years. Trial participants from South Africa and Uganda (72 per each country) were randomized into 9 different arms (**Figure 1**), with 16 individuals included in each trial arm (8 per each country). Arm 1 comprised control individuals who received no PrEP, and the eight additional arms allocated individuals to drugs (FTC-TDF or FTC-TAF), number of doses and tablets (2 at dose 1; 1 at dose 2) and interval from the last dose (5 or 21 hours) to circumcision after PrEP administration.

**Table 1** shows trial participant baseline characteristics, stratified by PrEP doses. All individuals were HIV negative and abstinence from sexual intercourses was not requested to be enrolled in the trial. Sexually transmitted infections (STIs) screens carried out at enrolment identified 6 cases of chlamydia. Participants were randomized into trial arms, therefore baseline characteristics for both measured and unmeasured variables are expected to be balanced between trial arms.

**Table 1:** Characteristics of study participants by number of PrEP doses.

Characteristic	Control (n=16)	FTC-TDF 1 dose (n=32)	FTC-TDF 2 doses (n=32)	FTC-TAF 1 dose (n=32)	FTC-TAF 2 doses (n=32)
Country					
South Africa	8 (50%)	16 (50%)	16 (50%)	16 (50%)	16 (50%)
Uganda	8 (50%)	16 (50%)	16 (50%)	16 (50%)	16 (50%)
Age, mean (SD)	17.9 (3.7)	18.5 (3.3)	18.7 (3.2)	19.4 (2.5)	18.7 (3.5)
Weight (kg), mean (SD)	53.4 (10.1)	54.5 (8.7)	56.7 (9.6)	59.8 (8.6)	59.8 (11.4)
Height (cm), mean (SD)	165.3 (11.8)	163.7 (9.6)	164.3 (9.4)	167.9 (7.7)	168.3 (10.8)
Currently studying					
No	10 (63%)	18 (56%)	18 (56%)	13 (41%)	18 (56%)
Yes	6 (38%)	14 (44%)	14 (44%)	19 (59%)	14 (44%)
Current relationship status					
Single	9 (56%)	16 (52%)	17 (53%)	16 (50%)	20 (63%)
Boyfriend/girlfriend	7 (44%)	14 (45%)	14 (44%)	16 (50%)	12 (38%)
Other	0 (0%)	1 (3%)	1 (3%)	0 (0%)	0 (0%)
Ever had sex					
No	8 (50%)	15 (48%)	11 (34%)	10 (31%)	11 (34%)
Yes	8 (50%)	16 (52%)	21 (66%)	22 (69%)	21 (66%)
Chlamydia test result					
Negative	16 (100%)	31 (97%)	31 (97%)	30 (94%)	30 (94%)
Positive	0 (0%)	1 (3%)	1 (3%)	2 (6%)	2 (6%)

### Foreskin preparation

Circumcision was performed using the dorsal slit method either with or without the aid of a clamp and foreskins were shipped within 1 h to the local laboratories in Uganda and South Africa for processing. Upon arrival to the laboratories in the respective countries, inner and outer foreskin tissues were separated to be processed in parallel; smaller, 5–7 mm^2^-sized sections were excised. Each experimental sample included in the present study contained one inner explant and one outer explant stored in the same tube with RNAlater and frozen at -80°C. Explants include all the layers from the epidermis [stratum corneum, keratin layer, stratum granulosum, stratum spinosum and stratum basale (14)] and dermis. Hence, tissue explants include epithelial cells, keratinocytes, fibroblasts, Langerhans cells, T cells, dendritic cells, macrophages (15, 16). The blood vessels present in the foreskin (17) were not dissected from the specimens.

Samples were kept at -80°C during and after transportation to the Karolinska Institutet until the analyses were conducted.

### RNA sequencing of foreskin tissue

Tissue samples were disrupted and homogenized using a Tissuelyzer (Qiagen) and total RNA isolated using the RNeasy Kit (Qiagen) according to manufacturer’s instructions. RNA was subjected to quality control with Agilent Bioanalyzer (Agilent). To construct libraries suitable for Illumina sequencing, the Illumina stranded mRNA prep ligation sample preparation protocol was used with starting concentration of 200 ng total RNA. The protocol includes mRNA isolation, cDNA synthesis, ligation of adapters and amplification of indexed libraries. The yield and quality of the amplified libraries were analysed using Qubit by (Thermo Fisher) and the Agilent Tapestation (Agilent). The indexed cDNA libraries were normalized and combined, and the pools were sequenced on the Illumina Novaseq 6000 S4 flowcell for a 150 cycles paired-end sequencing run generating 150 bp paired-end reads. A single tissue sample was obtained for each participant included in the study from which transcriptome was derived.

### RNA-seq data processing and analysis

Sample demultiplexing was performed using bcl2fastq (v2.20.0, Illumina), and quality and adapter trimming of reads was performed using Cutadapt (v2.8). Sample quality was assessed using FastQC (v0.11.8) and MultiQC (v1.7). Reads were aligned to the Ensembl GRCh38 reference genome using STAR (v2.6.1d). Counts for each gene were obtained using featureCounts (v1.5.1).

### Study approval

Ethical clearance to conduct the trial was obtained from the South African Health Products Regulatory Authority (Ref: 20181004); the Uganda Virus Research Institute research ethics committee (GC/127/18/12/680); Uganda National Council of Science and Technology (HS 2534); Uganda National Drug Authority (618/NDA/DPS/09/2019) and the London School of Hygiene and Tropical Medicine research ethics committee (Ref:17403). Informed written consent was obtained from all participants. The Swedish Ethics Review Authority approved the laboratory studies of the collected specimens at the Karolinska Institutet (2020–00941).

### Data analysis

Bioconductor package DESeq2 (v1.22.2) (18) was used for normalization and sample group comparisons, generating log_2_ fold-changes, Wald test p-values and p-values adjusted for multiple testing (Benjamini-Hochberg method, FDR). One outlier, defined by a z-score > 3, was excluded from the analysis. Volcano plots used for the graphical presentation of differentially expressed genes (DEGs; adj.p < 0.05, log_2_FC > 1) were constructed using EnhancedVolcano. To perform principal component analysis (PCA) gene counts were first transformed using the *vst* function (part of the DESeq2 package); the *plotPCA* function was then used to calculate principal components (18). Gene ontology (GO) terms association were acquired using the *groupGO* function from clusterProfiler (19) by extracting the GO term at a level where only a single term is related to the gene of interest. GO terms were then used as input for the web interface of REVIGO (20), which visualized them as semantic space scatter plots. DEGs identified in the groups receiving FTC-TDF/FTC-TAF were used to perform functional enrichment analysis using the *gost* function of g:Profiler (21) with default parameters.

## Results

### Differentially expressed genes in FTC-TDF and FTC-TAF arms

**Figure 2:**
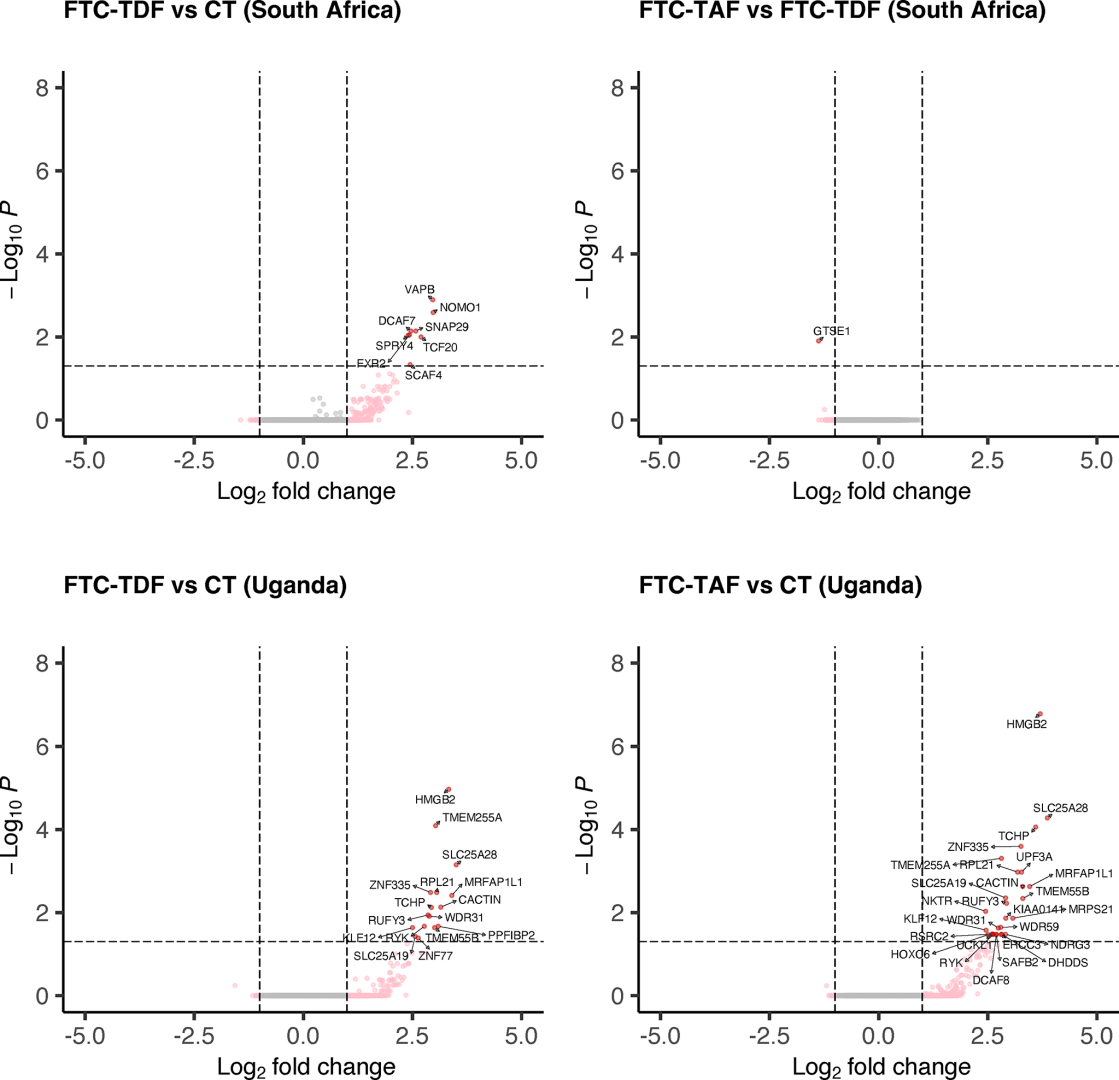
Differential gene expression analysis of South African and Ugandan cohorts. Individuals from South Africa and Uganda were stratified according to the type of administered PrEP drug and the expression of transcripts was compared to the corresponding control arm or between drug groups. Only comparisons with statistically significant (p < 0.05) differentially expressed genes (protein-coding, N=15378) are shown for each country.

PrEP arms from Uganda and South Africa were analyzed separately according to drug (FTC-TDF arms 2-5 and FTC-TAF arms 6-9) and compared to the corresponding local control arm 1 (**Figure 2**). This comparison comprises all protein coding genes (N=15378).

In South Africa, the comparison of the FTC-TAF arms with controls revealed no DEGs whereas treatment with FTC-TDF resulted in the modulation of eight genes (*SCAF4, TCF20, SPRY4, FXR2, DCAF7, SNAP29, NOMO1, VAPB*; **Figure 2**). Grouping of the genes by biological processes revealed that five of these genes (*FRX2, SCAF4, TFC20, SPRY4, VAPB*) were involved in metabolic functions that included cellular response to stimulus. Interestingly, the comparison of transcriptomes from foreskins obtained from individuals receiving either FTC-TAF or FTC-TDF revealed only 1 down-regulated DEG, *GTSE1*, which regulates cell cycle transition.

In foreskin specimens from Uganda the comparison between drugs revealed no DEGs. On the other hand, several DEGs were detected when comparing FTC-TAF to control (n=27) and FTC-TDF to control (n=16) (**Figure 2**). Similar to the changes observed in the South African cohort, the DEGs were involved in various metabolic processes (FTC-TAF: 17/27 DEGs; FTC-TDF: 9/16 DEGs) as well as in the response to stimulus (FTC-TAF: 9/27 DEGs; FTC-TDF:4/16 DEGs). The two comparisons FTC-TAF and FTC-TDF versus controls shared the following DEGs: *HMGB2*, *SLC25A28*, *TCHP*, *ZNF335*, *TMEM255A*, *RPL21*, *MRFAP1L1*, *CACTIN*, *SLC25A19*, *TMEM55B*, *RUFY3*, *WDR31*, *KLF12* and *RYK* (n=14). Information on the proteins coded by these genes is presented in **Supplementary Table 1**.

**Figure 3:**
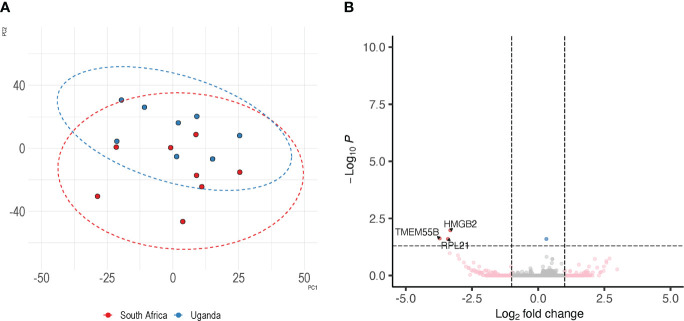
Similarity of control specimens in Uganda and South Africa. To evaluate the extent of inter-country variability we performed a differential expression analysis between the control arms of South Africa and Uganda. Panel **(A)** depicts the distribution of control specimens produced by PCA; the overlap between the two countries is shown by corresponding ellipse. Panel **(B)** shows the detection of 3 DEGs between the controls from the two trial sites.

The limited size of the control arms could possibly cause DEGs to be identified as significantly different in the comparisons with PrEP arms due to a skewed random selection of individuals included in the control arms and not as result of PrEP. In order to minimize the chances of this possible bias we compared the control arms from the two countries to evaluate the possibility of merging transcriptome data from controls and individuals receiving PrEP at the two sites. Principal component analysis (PCA) revealed that the control groups from the two countries are contiguous and, to some extent, overlapping (**Figure 3A**). Additionally, a differential expression analysis of the Uganda versus South Africa control groups revealed only three down-regulated DEGs in Uganda (*HMGB2*, *TMEM55B*, *RPL21*) (**Figure 3B**; **Supplementary Table 1**).

This analysis, with the few differences identified between the two control arms, supported our decision to merge PrEP and control arms from the two countries.

**Figure 4:**
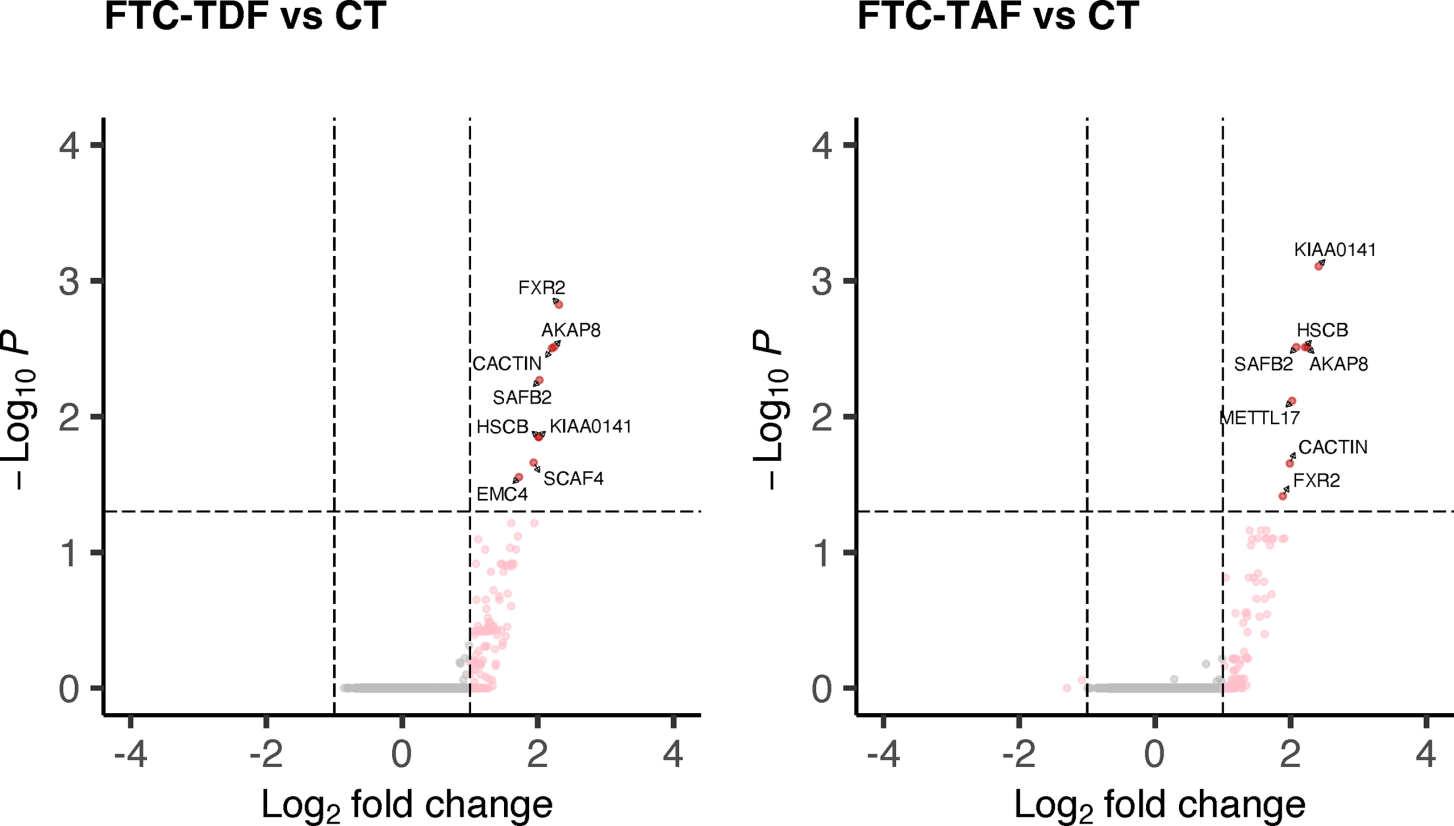
DEGs in merged PrEP-treatment arms as compared to control arm. All specimens from South Africa and Uganda were merged and stratified by administered PrEP drug. The merged arms were then compared to the control group and the statistically significant DEGs shown according to the administered PrEP drug.

Using this approach, we identified 8 DEGs in the comparison FTC-TDF versus controls and seven DEGs in the comparison FTC-TAF versus controls (**Figure 4**; **Supplementary Table 1**); interestingly *KIAA0141*, *FXR2*, *AKAP8*, *SAFB2*, *HSCB*, *CACTIN*, were upregulated by both drugs. In addition, *METTL17* was upregulated only in the comparison FTC-TAF versus controls; *EMC4* and *SCAF4* were upregulated only in the comparison FTC-TDF versus controls. It is of note that the majority of these genes are involved in innate immunity (*AKAP8*, *CACTIN*) as well as cellular stress response (*KIAA0141*, *HSCB*, *METTL17*, *EMC4*) (**Supplementary Table 1**).

### Number of PrEP doses affects foreskin transcriptome changes

**Figure 5:**
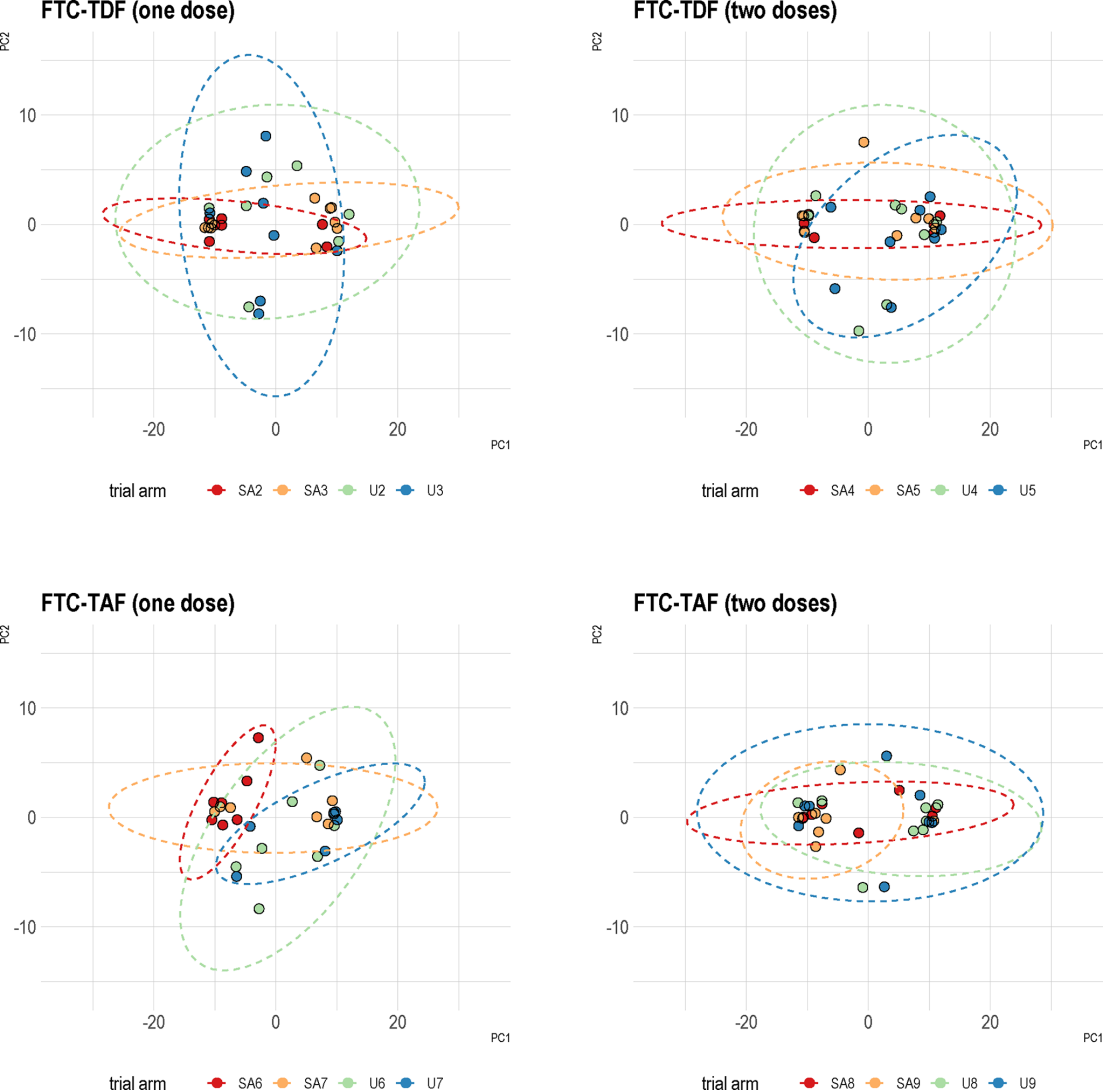
Principal component analysis of Uganda and South Africa trial arms combined together. Specimens from the two trial sites were analyzed based on the type of PrEP and number of administered doses. PCA was then performed using protein coding transcripts (N=15378). The overlap between the trial arms from two countries is shown by corresponding ellipses. A color coding in the figure denotes the different trial arms.

We used PCA to investigate the similarity between arms which received the same drug dose but with different time to circumcision in the two countries (arms 2 and 3; 4 and 5; 6 and 7; 8 and 9). The PCA analysis showed that samples from individuals receiving the same drug dose are similar and do not separate into homogenous clusters (**Figure 5**).

**Figure 6:**
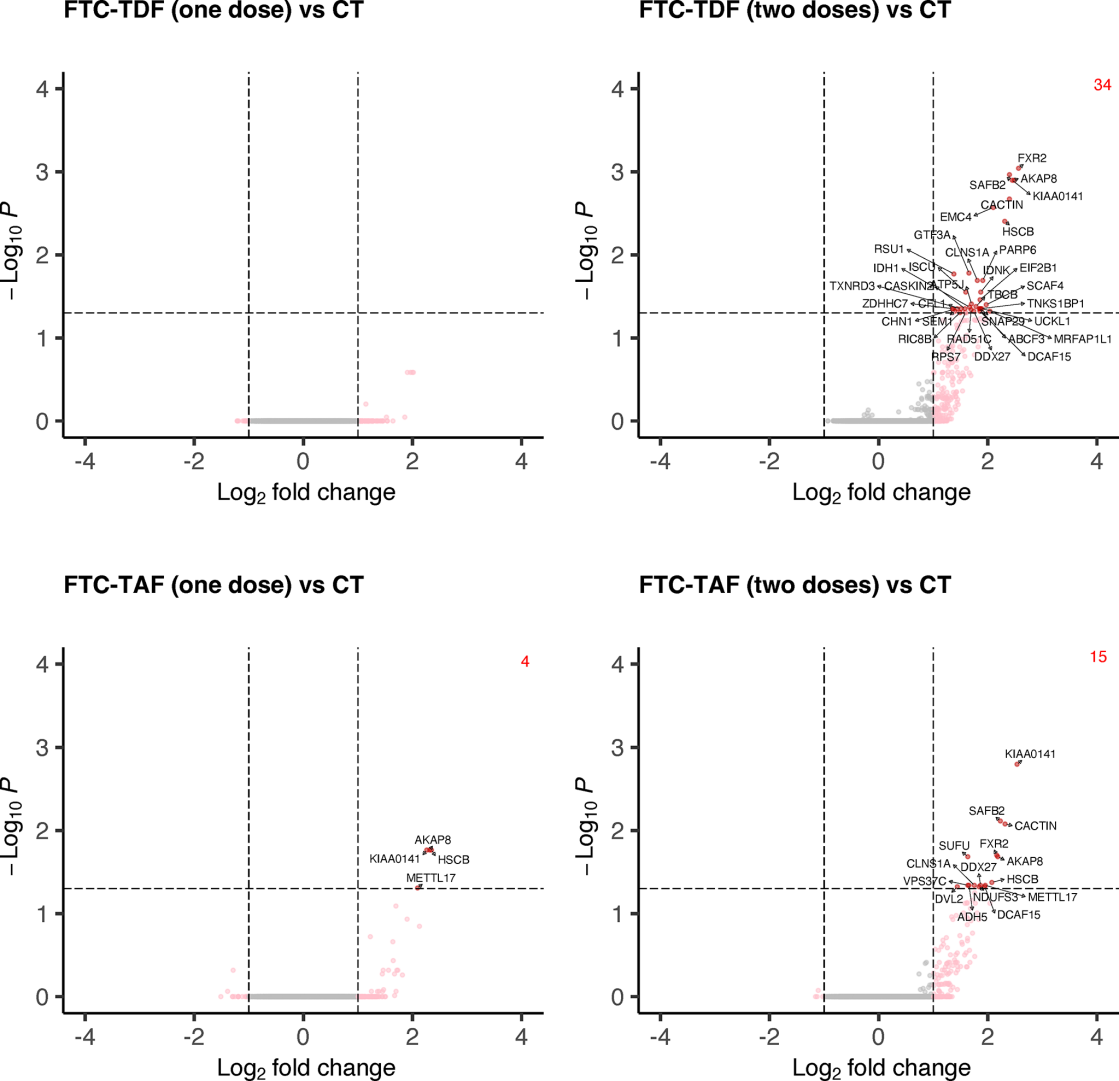
Differential gene expression analysis comparing merged PrEP arms receiving different drugs and doses with control arm. Specimens from both South Africa and Uganda were grouped by the type of PrEP and number of administered doses. The expression of all transcripts was then compared to the control arm and statistically significant DEGs and their number are shown in red.

Accordingly, to elucidate the impact of PrEP dosing on the transcriptome sequences of foreskin tissues we performed differential expression analysis of the merged cohorts from Uganda and South Africa according to the number of doses (one or two doses) of the two different PrEP drugs (FTC-TDF or FTC-TAF) in comparison with merged control arms (**Figure 6**).

One dose of FTC-TDF did not affect the foreskin transcriptome in relation to control arm; one dose of FTC-TAF revealed an upregulation of four DEGs (*AKAP8*, *KIAA0141*, *HSCB*, *METTL17*). On the other hand, two doses of either FTC-TDF or FTC-TAF significantly increased the number of induced DEGs; in common to the two drugs were *KIAA0141*, *SAFB2*, *CACTIN*, *FXR2*, *AKAP8*, *HSCB*, *CLNS1A*, *DDX27* and *DCAF15*. In addition, two doses of FTC-TAF induced the up-regulation of six more DEGs (**Supplementary Table 2**); two doses of FTC-TDF induced the upregulation of additional 25 genes (**Supplementary Table 2**). We analyzed the effect of PrEP dose escalation (one or two doses) of FTC-TAF and FTC-TDF on expression of the DEGs described above. Interestingly, we observed a trend of upregulation after two doses of either drug (**Supplementary Figure 1**). The DEGs identified when comparing PrEP to control groups (**Figure 6**) were used to perform functional enrichment analysis by g:profiler. The result of these analyses is presented in **Supplementary Tables 3**, **4** where it is shown that there were no significantly enriched biological processes. The analyses mostly revealed associations with cellular/biological compartments as evidenced by the “GO : CC-cellular compartments” and “HPA-Human protein Atlas” results.

We also analyzed whether age variation within the groups of individuals who received one or two doses of either FTC-TDF or FTC-TAF, or the control arm, could impact on the outcome of analyses once the specimens collected from Uganda and South Africa were analyzed together. In **Supplementary Figure 2** volcano plots show the comparisons of foreskin transcripts from adolescents (up to 19 years of age) and adults (>19 years) within the drug groups, or the control arm. No DEGs related to the comparisons adolescents versus adults were identified for the groups 1xFTC-TAF, 1xFTC-TDF, 2xFTC-TAF, 2xFTC-TDF; two DEGs, ACKR4 and COROB2, were identified in the control arm.

**Figure 7:**
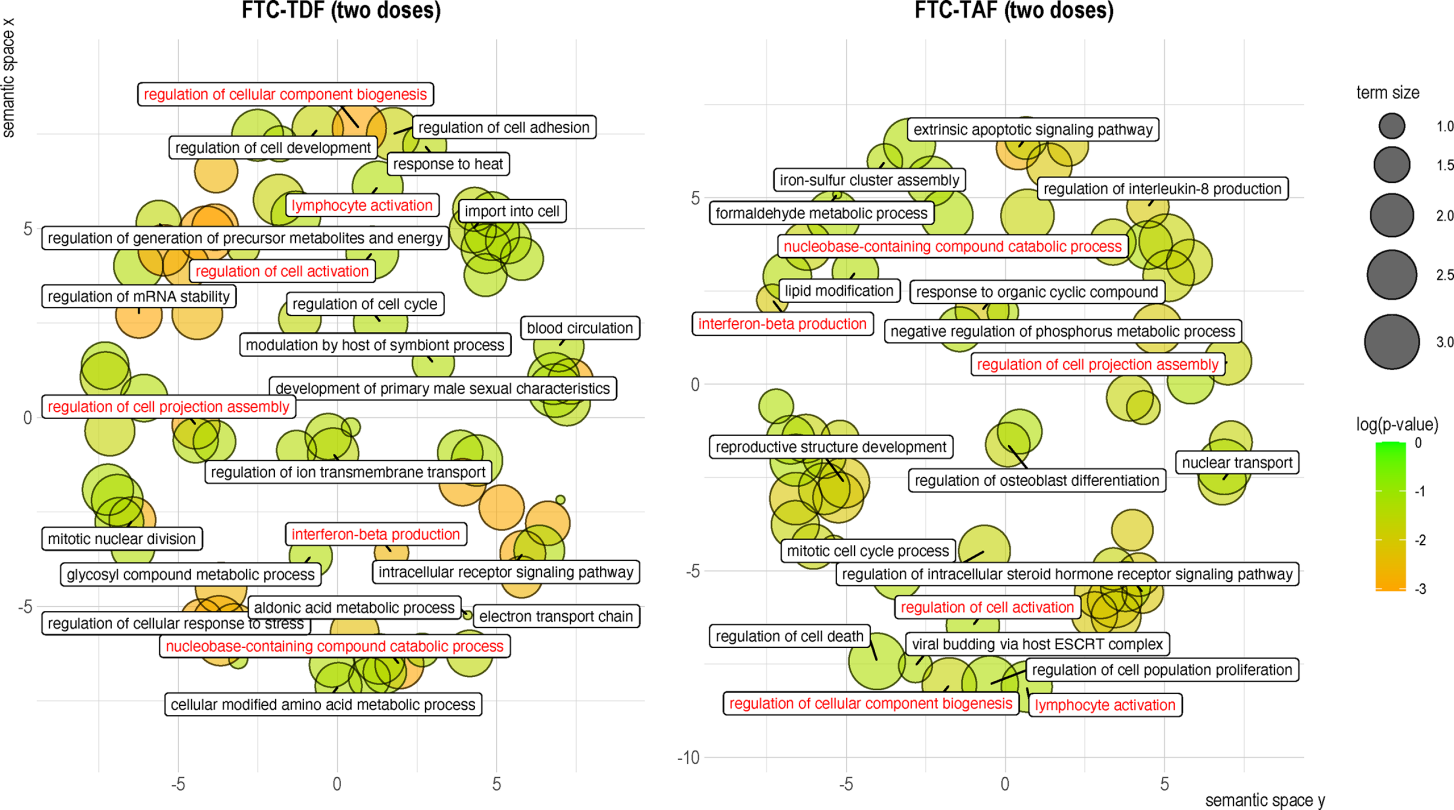
Clustering of gene ontology terms associated with DEGs in the merged drug and dose arms. GO associations with DEGs were established by using clusterProfiler to extract a single GO term for each DEG. The biological process GO terms associated with each of the identified DEGs were then analyzed using REVIGO and the semantic space clusters visualized for each PrEP regimen. GO terms are represented by circles, whose size indicates the relative number of genes included in the term. Circles are colored according to the significance (p-value) of the gene they represent. Labels correspond to the GO term determined to be representative of the cluster. Shared GO terms are shown in red.

To further clarify the functional involvement of the DEGs identified in arms treated with different drugs and doses we extracted biological process GO terms associated with each DEG and then used the REVIGO package to cluster them by semantic similarity (**Figure 7**). One dose of FTC-TAF, which resulted in the significant modulation of four genes produced few and small functional clusters (not shown). Analysis of the DEGs discovered after two doses of FTC-TDF revealed that this PrEP regimen most significantly affected biological processes related to the cellular response to stress, regulation of cellular component biogenesis, generation of metabolites and energy, intracellular signaling and mRNA stability among others (**Figure 7**). In foreskin specimens obtained after two doses of FTC-TAF, we observed a significant impact of biological processes such as apoptotic signaling, reproductive structure development, regulation of steroid hormone signaling and regulation of interleukin-8 production (**Figure 7**). Interestingly, two doses of either FTC-TDF or FTC-TAF impacted the expression of genes which were shared and associated with several function (shown in red in **Figure 7**), including: regulation of cell activation, lymphocyte activation, interferon beta production, regulation of cell projection assembly, regulation of cellular component biogenesis and nucleobase-containing compound catabolic process.

## Discussion

In the present study we evaluated the impact of short-term oral PrEP on the transcriptome of human foreskin tissue from participants of the CHAPS study that was conducted in Uganda and South Africa. The present study is, to our knowledge, the first to evaluate the impact of short-term oral PrEP on foreskin transcriptome in young men following one to two days administration of FTC-TDF or FTC-TAF. Our results showed that one dose of FTC-TDF or FTC-TAF modestly affected the foreskin transcriptome, whereas two daily doses of FTC-TDF or FTC-TAF produced a readily detectable array of modulated genes involved in inflammation, mitochondrial function and cell proliferation. We also demonstrate that two daily doses of either PrEP modality results in a significant increase of differentially expressed genes.

Our findings concur with other studies investigating the effect of TFV-based drugs on the transcriptome of healthy individuals (8, 10, 22). In our analyses, the combined differences of either FTC-TDF or FTC-TAF amounted to nine genes, all of which were upregulated. A finer stratification of the specimens taking into account different doses of each PrEP drug revealed 40 upregulated genes following administration of two PrEP doses. While the study by Hladik et al., which evaluated gene expression after application of 1% TFV gel on the rectal mucosa, reported a prevailing suppressive effect of seven daily applications, their results pointed out that a single application does in fact produce a predominant gene induction consisting of 70 upregulated and six downregulated genes (8). Pharmacokinetics should also be considered when comparing the extent of transcriptomic changes caused by PrEP. Topical application of TFV is known to result in significantly higher drug levels in tissue as compared to oral administration (10), which is likely why we observed fewer DEGs comparing with the trials using 1%TFV gel. Indeed, a confined transcriptomic response has been reported when PrEP was administered orally in women even over periods longer than our study utilized (22).

Inflammation is of particular importance as a risk factor for HIV-1 acquisition as cellular activation increases the susceptibility of target cells to becoming infected. Our results did not indicate any agonistic effect of inflammatory responses after PrEP administration. We found, however, that several genes including *CACTIN* (coding for the Cactin protein) and *AKAP8* (A-Kinase Anchoring Protein 8), which are involved in the orchestration of inflammatory signaling, were modulated. Overexpression of *CACTIN*, which was significantly upregulated in our study, has been described to inhibit TLR-induced activation of NF-κB and interferon-regulatory transcription factors as well as the induction of TLR-responsive genes (23). All known TLRs except TLR3 activate NF-κB, which in turn leads to the production of a plethora of pro-inflammatory cytokines (24). Moreover, TLR7 and TLR8 (25–27) respond to HIV-1 infection by triggering type I IFN (28) or interleukin-1b production (29); stimulation of both these receptors has been shown to lead to increased HIV-1 replication in productively infected cells (30), whereas their inhibition has the opposite effect (31) suggesting that the dampening of NF-κB activity mediated by the *CACTIN* upregulation which we observed in the present study could function to suppress viral replication. In addition, our results show that *AKAP8*, which is also involved in TLR signaling, was upregulated by both PrEP modalities. Upon their ligation, TLRs enhance the production of prostaglandin E_2_ (PGE_2_), whose immunomodulatory functions stem from its capacity to activate cAMP signaling (32, 33). Acting downstream of PGE_2_, *AKAP8* facilitates cAMP-mediated inhibition of TNF-α (32) possibly promoting an anti-inflammatory effect.

The antiviral activity of nucleoside reverse transcriptase inhibitors (NRTIs) is attributed to their ability to disrupt the function of the viral DNA polymerase, reverse transcriptase (7, 34). Because of its structural similarity to reverse transcriptase, the function of mitochondrial DNA polymerase γ, which is responsible for replication of mitochondrial DNA, can also be disrupted by NRTIs (35). Due to this, mitochondrial toxicity is another concern associated with the use of NRTIs such as TFV. Our results indicate that administration of both FTC-TDF and FTC-TAF resulted in the modulation of several genes that reflect the state of mitochondrial homeostasis. The protein DAP3 Binding Cell Death Enhancer 1, encoded by *KIAA0141*, which was upregulated after two doses of FTC-TDF and FTC-TAF PrEP regimens, localizes in the mitochondria and is released to the cytosol in the event of stress-related mitochondrial perturbation. In the cytosol, *KIAA0141 *product activates a signaling pathway, which culminates in the phosphorylation of eIF2α triggering the integrated stress response (ISR) (36, 37). The gene *EMC4*, that was differentially expressed after FCT-TDF administration, codes for the protein ER Membrane Protein Complex Subunit 4, which is heavily involved in biogenesis of the mitochondrial membrane by facilitating the transfer of phospholipids from the endoplasmic reticulum (38) and plays a role in ISR by stabilizing initiation factor eIF-2B subunit alpha, which regulates protein translation during stress conditions (39). Further strengthening the possibility of PrEP causing mitochondrial stress is the fact that our PrEP dose-dependent analysis revealed the gene of the alpha subunit of eIF-2B to be significantly upregulated after two doses of FTC-TDF. Finally, the dysregulated expression of the protein products of *HSCB* (upregulated after both PrEP modalities) a chaperone in iron-sulfur cluster assembly in mitochondria and *METTL17* (upregulated after FTC-TAF), a regulator of mitochondrial ribosomal RNA modifications required for translation of mitochondrial coding genes, suggest an impact of PrEP on mitochondrial functions (40).

The mitochondrial impact of PrEP has been examined by Bowman et al. (41), who cultured human peripheral blood mononuclear cells (PBMCs) from healthy donors in FTC-TDF for 24 hours. Analysis of essential parameters such as mitochondrial basal and maximum respiration, spare respiratory capacity, ATP production, proton leak and nonmitochondrial respiration revealed an overall decrease in oxygen consumption rate kinetics in PrEP-treated PBMCs. Additionally, monocytes and CD4+/CD8+ T cells exposed to FTC-TDF were found to have a significantly reduced mitochondrial mass and an increased level of reactive oxygen species, likely a direct result of mitochondrial dysfunction (41). The possibility of analyzing mitochondrial perturbation induced by PrEP using morphological approaches should be considered in the future. We show that short-term usage of PrEP induces dysregulation of genes involved in mitochondrial function; additional studies should be performed in individuals utilizing PrEP on a long-term basis to evaluate if expression of genes involved in mitochondrial function is affected. It is however important to underline in this context that the PrEP clinical benefits outweigh the risks. As NRTI-sparing regimens become increasingly available, it is important to understand differences in toxicities between regimes.

Injury to mucosal tissue represents another concern when using PrEP as it can affect adherence, reduce efficacy and more importantly, even increase the risk of HIV-1 acquisition. Several studies have linked disruption of epithelial integrity and inflammatory processes to poor protection against HIV-1 transmission (42–44). This issue has been particularly associated with topical TFV-based solutions that tend to result in a high drug concentration in tissue. Indeed, a study evaluating the impact of 1% TFV gel on the rectal mucosa proteome after eight daily applications identified 17 factors after one and 25 factors after eight applications with a significantly altered abundance in treated tissue (9). Of these, 13 proteins, which included epithelial integrity factors, serpins and cytoskeletal proteins could distinguish TFV from placebo groups with 100% accuracy. Our study did not reveal any genes associated with epithelial integrity to be modulated after one or two PrEP doses, which suggests that oral administration represents a safer PrEP modality.

Our study has a few limitations. First, the CHAPS clinical trial was designed for the purpose of investigating the pharmacodynamics and pharmacokinetics of two different PrEP drugs and as such required numerous arms. To recruit a sufficient number of participants the trial was conducted in two distinct geographical locations with the trial arms being merged for the endpoint analyses. Although differential expression analysis of the control arms revealed very few DEGs and thus justified merging, the possibility remains that environmental factors specific to the two countries may have contributed to divergent response to treatment and affected our results. A larger scale study with a more homogenous sample size could address this issue. Second, the trial was not specifically powered to detect differences in transcriptome profile; it is thus possible that the sample size was not sufficient to detect important differences between trial arms, and therefore our results should be considered as exploratory. To compensate for this possibility, we have grouped foreskin samples from individuals receiving the same PrEP dose and in addition pooled samples from the two countries. Third, as a part of the trial design we only investigated the impact of short-term PrEP administration. The effect of prolonged PrEP use on the foreskin mucosa has yet not been studied but is important to explore in future studies. Finally, in our study we used foreskin tissue to perform bulk RNA sequencing, which includes various cell types and may mask differences in important, but rare immune cells. Separate analyses of inner and outer foreskin tissue and targeted sequencing of selected cell types may reveal further clues about the processes affected by PrEP administration.

In summary, the results presented in this study indicate that the use of short term on-demand PrEP does not induce inflammatory responses in the foreskin tissue and that the expression of genes involved in foreskin tissue integrity are not affected during oral administration of FTC-TDF and FTC-TAF. We however describe an upregulated expression of genes involved in diverse mitochondria-related functions. Although it is difficult to predict whether these changes in gene expression result in an overall worsened mitochondrial function, additional studies need to be conducted to assess that this is not the case. In this context it is also important that in the study conducted by Hladik et al. (8) on topical application of 1% TFV gel on the rectal mucosa a dysregulated expression of genes involved in mitochondrial function was reported. Overall, our data show that short-course on-demand oral PrEP in men is not a benign formulation but modulates genes in foreskin tissue which are likely unfavorable to HIV replication and potential acquisition. Further studies are warranted that would examine long-term use of oral FTC-TDF and FTC-TAF PrEP in men and how changes in foreskin tissue offer an additional mechanism, over and above their anti-viral properties, to the effectiveness of these drugs in preventing HIV-1 infection.

## Data availability statement

The data presented in the study are deposited in the GenBank Data Libraries repository, accession number PRJNA884284.

## Ethics statement

The studies involving human participants were reviewed and approved by ethical clearance to conduct the trial was obtained from the South African Health Products Regulatory Authority (Ref: 20181004); the Uganda Virus Research Institute research ethics committee (GC/127/18/12/680); Uganda National Council of Science and Technology (HS 2534); Uganda National Drug Authority (618/NDA/DPS/09/2019) and the London School of Hygiene and Tropical Medicine research ethics committee (Ref:17403). Informed written consent was obtained from all participants. The Swedish Ethics Review Authority approved the laboratory studies of the collected specimens at the Karolinska Institutet (2020–00941). Written informed consent to participate in this study was provided by the participants’ legal guardian/next of kin.

## Author contributions

SP: Conducted experiments, analyzed the data, created figures, wrote the manuscript. CH: Planned the study, conducted experiments, analyzed the data, wrote the manuscript. LE: Conducted experiments, analyzed the data. LL, DO, AS: Selected trial participants, obtained signed informed consent and clinical specimens. TS, A-DP, SM, PN and GO: Sample processing, conducted experiments. JS: Conducted experiments and analyzed the data. PK: Planned the study. EW: Analyzed data and wrote the paper. SK: Planned study and analyzed specimens. NM, CG and JF: Planned the study, wrote the manuscript. FC: Planned the study, analyzed the data, wrote the manuscript. All authors contributed to the article and approved the submitted version.

## Funding

This study is part of the EDCTP2 programme supported by the European Union (grant number RIA2016MC-CHAPS). The study received support by a grant from the Swedish Research Council (Francesca Chiodi; Vetenskapsrådet 2019-04596).

## Acknowledgments

We would like to thank the core facility at NEO, BEA, Bioinformatics and Expression Analysis, which is supported by the board of research at the Karolinska Institute and the research committee at the Karolinska Hospital.

## Conflict of interest

The authors declare that the research was conducted in the absence of any commercial or financial relationships that could be construed as a potential conflict of interest.

## Publisher’s note

All claims expressed in this article are solely those of the authors and do not necessarily represent those of their affiliated organizations, or those of the publisher, the editors and the reviewers. Any product that may be evaluated in this article, or claim that may be made by its manufacturer, is not guaranteed or endorsed by the publisher.

## Supplementary material

Supplementary Figure 1 Effect of PrEP dose escalation on gene expression. The fold change expression relative to control group of significant DEGs in is shown after one and two doses of FTC-TAF panel **(A)** and FTC-TDF panel **(B)**.

Supplementary Figure 2 Impact of age on gene expression according to PrEP regimen. Treatment and control groups consisting of specimens collected from Uganda and South Africa were divided according to age into adolescents (up to 19 years of age) and adults (>19 years). Vulcano plots show the comparisons of foreskin transcripts within the drug groups, or the control arm. No DEGs related to the comparisons adolescents versus adults were identified for the groups 1xFTC-TAF, 1xFTC-TDF, 2xFTC-TAF, 2xFTC-TDF; two DEGs were identified in the control arm.
