# Supplementary figures and images for: Short-term oral pre-exposure prophylaxis against HIV-1 modulates the transcriptome of foreskin tissue in young men in Africa
Source: Front Immunol. 2022 Nov 18;13:1009978. doi: 10.3389/fimmu.2022.1009978 (PMC9720390; doi:10.3389/fimmu.2022.1009978)

**A**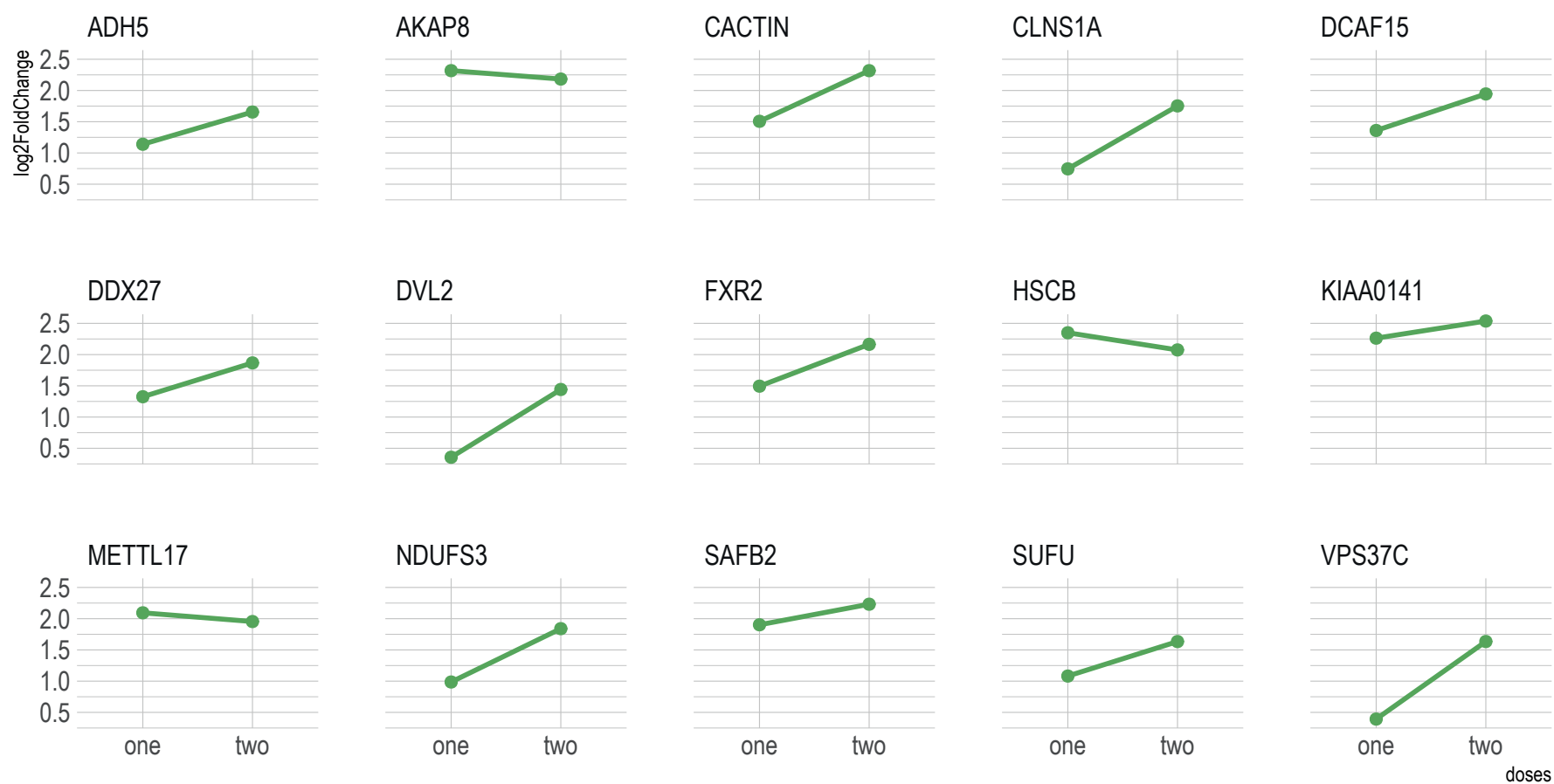**B**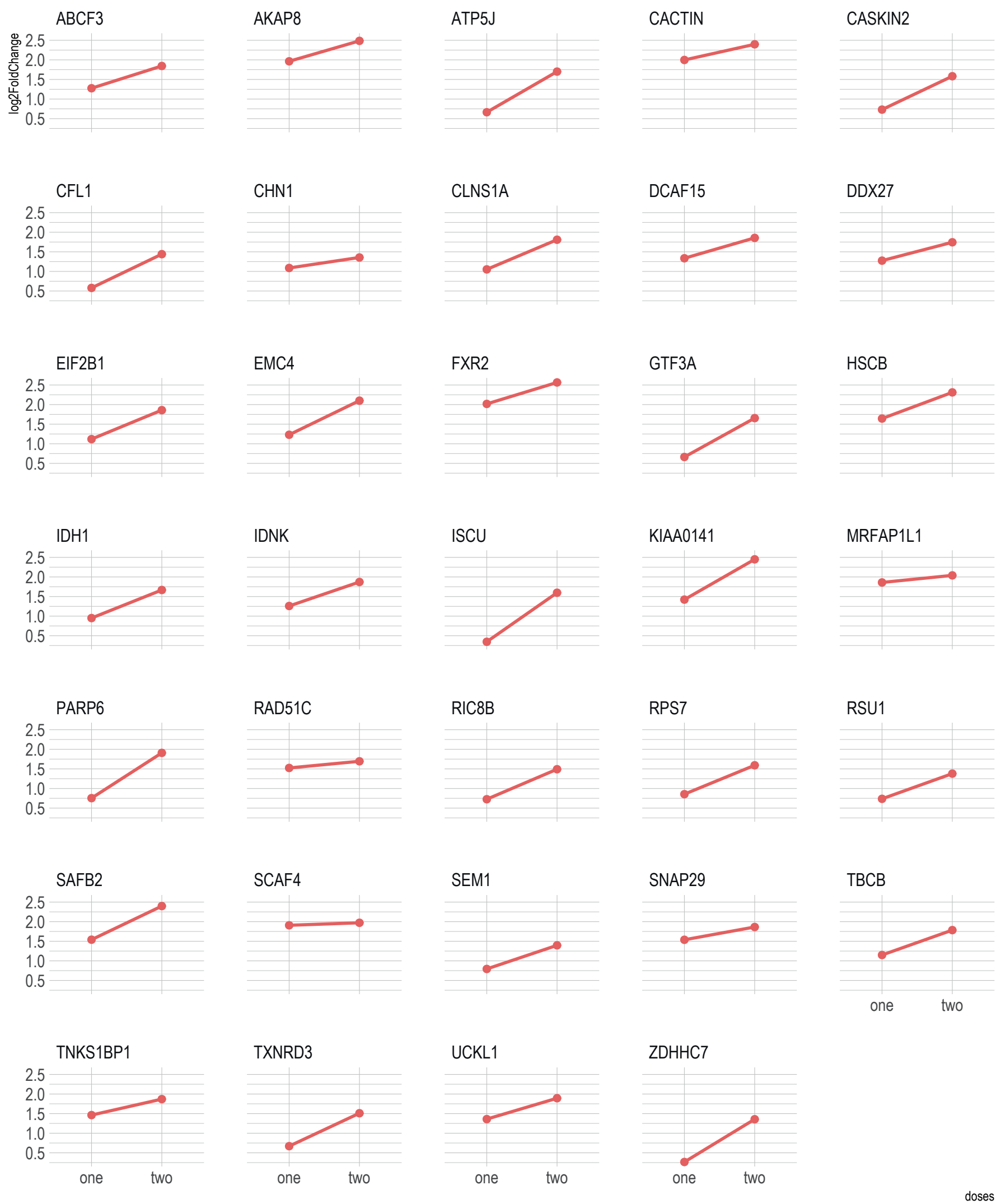

Supplement: Supplementary Figure 1 — Effect of PrEP dose escalation on gene expression. The fold change expression relative to control group of significant DEGs in is shown after one and two doses of FTC-TAF panel (A) and FTC-TDF panel (B). [file Image_1.pdf]
