# Supplementary material for: Short-term oral pre-exposure prophylaxis against HIV-1 modulates the transcriptome of foreskin tissue in young men in Africa
Source: Front Immunol. 2022 Nov 18;13:1009978. doi: 10.3389/fimmu.2022.1009978 (PMC9720390; doi:10.3389/fimmu.2022.1009978)
Supplement: Supplementary Figure 2 — Impact of age on gene expression according to PrEP regimen. Treatment and control groups consisting of specimens collected from Uganda and South Africa were divided according to age into adolescents (up to 19 years of age) and adults (>19 years). Vulcano plots show the comparisons of foreskin transcripts within the drug groups, or the control arm. No DEGs related to the comparisons adolescents versus adults were identified for the groups 1xFTC-TAF, 1xFTC-TDF, 2xFTC-TAF, 2xFTC-TDF; two DEGs were identified in the control arm. [file Image_2.pdf]

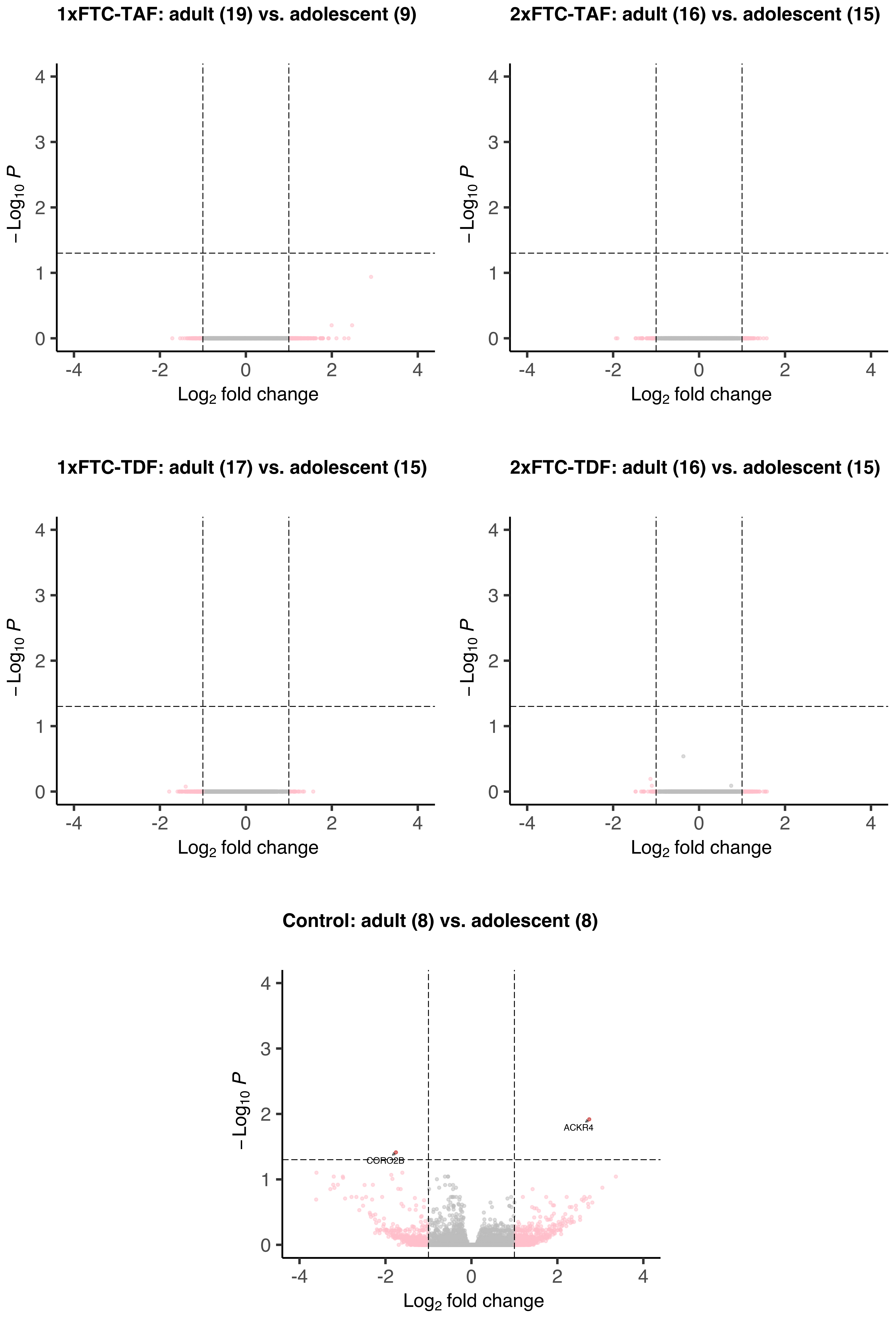
1xFTC-TAF: adult (19) vs. adolescent (9)
2xFTC-TAF: adult (16) vs. adolescent (15)
1xFTC-TDF: adult (17) vs. adolescent (15)
2xFTC-TDF: adult (16) vs. adolescent (15)
Control: adult (8) vs. adolescent (8)
−Log10 P
Log2 fold change
CORO2B
ACKR4
