# Supplementary material for: Short-term oral pre-exposure prophylaxis against HIV-1 modulates the transcriptome of foreskin tissue in young men in Africa
Source: Front Immunol. 2022 Nov 18;13:1009978. doi: 10.3389/fimmu.2022.1009978 (PMC9720390; doi:10.3389/fimmu.2022.1009978)
Supplement: Supplementary file 5 [file Table_3.pdf]

Supplementary table 3. Functional enrichment analysis of DEGs (n= 34) in participants receiving two doses of FTC-TDF.

id	source	term_id	term_name	term_size	intersection_size		p_value
1	GO:CC	GO:0005829	cytosol	5420	20		3.9e-04
2	HPA	HPA:0130241	colon; peripheral nerve/ganglion[≥Low]	5017	21		4.5e-03
3	HPA	HPA:0510000	soft tissue 2	5178	21		8.1e-03
4	HPA	HPA:0460671	skin 1; melanocytes[≥Low]	5276	21		1.1e-02
5	HPA	HPA:0500000	soft tissue 1	5337	21		1.4e-02
6	HPA	HPA:0480052	small intestine; glandular cells[≥Medium]	6035	22		2.0e-02
7	HPA	HPA:0340461	ovary; ovarian stroma cells[≥Low]	3965	18		2.1e-02
8	HPA	HPA:0150052	duodenum; glandular cells[≥Medium]	6074	22		2.3e-02
9	HPA	HPA:0400052	rectum; glandular cells[≥Medium]	6077	22		2.3e-02
10	HPA	HPA:0460652	skin 1; fibroblasts[≥Medium]	2824	15		2.7e-02
11	HPA	HPA:0510651	soft tissue 2; fibroblasts[≥Low]	4106	18		3.5e-02
12	HPA	HPA:0130201	colon; endothelial cells[≥Low]	5649	21		4.0e-02
13	HPA	HPA:0130051	colon; glandular cells[≥Low]	6962	23		4.6e-02
14	HPA	HPA:0510652	soft tissue 2; fibroblasts[≥Medium]	2600	14		4.9e-02
15	HPA	HPA:0570743	testis; cells in seminiferous ducts[High]	845	8		5.0e-02
16	TF	TF:M09641_1	Factor: NRF-1; motif: SYGCGCMTGCGCRNNGSN; match class: 1	1005	11		1.0e-03
17	TF	TF:M07052_1	Factor: NRF-1; motif: GCGCMTGCGCN; match class: 1	745	9		6.3e-03

g:Profiler (biit.cs.ut.ee/gprofiler)
