# Supplementary material for: Short-term oral pre-exposure prophylaxis against HIV-1 modulates the transcriptome of foreskin tissue in young men in Africa
Source: Front Immunol. 2022 Nov 18;13:1009978. doi: 10.3389/fimmu.2022.1009978 (PMC9720390; doi:10.3389/fimmu.2022.1009978)
Supplement: Supplementary file 6 [file Table_4.pdf]

Supplementary table 4. Functional enrichment analysis of DEGs (n= 15) in participants receiving two doses of FTC-TAF.

id	source	term_id	term_name	term_size	intersection_size		p_value
1	GO:CC	GO:0005654	nucleoplasm	4215	9		3.8e-02
2	GO:CC	GO:0005739	mitochondrion	1683	6		4.7e-02
3	HPA	HPA:0241102	heart muscle; cardiomyocytes[≥Medium]	4348	11		4.7e-03
4	HPA	HPA:0570743	testis; cells in seminiferous ducts[High]	845	6		8.4e-03
5	HPA	HPA:0130202	colon; endothelial cells[≥Medium]	3693	10		1.8e-02
6	HPA	HPA:0010012	adipose tissue; adipocytes[≥Medium]	2219	8		3.2e-02
7	HPA	HPA:0460671	skin 1; melanocytes[≥Low]	5276	11		3.9e-02
8	HPA	HPA:0490692	smooth muscle; smooth muscle cells[≥Medium]	3114	9		4.5e-02
9	KEGG	KEGG:05217	Basal cell carcinoma	62	2		3.3e-02
10	TF	TF:M12642	Factor: NFYA; motif: NNRGCCAATSRGMRSSNNSN	2837	10		8.4e-03
11	TF	TF:M08692_1	Factor: ERF:pitx1; motif: NNCGGAWGNNNRGNTTA; match class: 1	894	6		4.7e-02

g:Profiler (biit.cs.ut.ee/gprofiler)
